# Molecular diagnostics using the QIAstat-Dx syndromic device for covering avian influenza pandemic preparedness

**DOI:** 10.1016/j.heliyon.2024.e40645

**Published:** 2024-11-26

**Authors:** Luis Peñarrubia, Sven Reister, Sara Jiménez-Guzmán, Roberto Porco, Clàudia Congost-Teixidor, Gemma Pueyo, Carla Camprubí-Font, Katariina Vara, Maria de la Cruz Cardenosa, Maria Contreras, Aida Mayorgas, Frederick van Deursen, Dietrich Lueerssen, Marti Juanola-Falgarona, Martin Schwemmle, Kevin Ciminski, Davide Manissero

**Affiliations:** a STAT-Dx Life S.L. (A QIAGEN Company) Baldiri Reixac, 4 Barcelona 08028, Spain; b QIAGEN GmbH, Qiagenstr.1 40724 Hilden, Germany; c QIAGEN Manchester Ltd, Citylabs 2.0, Hathersage Road, Manchester, M13 0BH, UK; d Institute of Virology, Medical Center University of Freiburg, Freiburg, Germany; e Faculty of Medicine, University of Freiburg, Freiburg, Germany

**Keywords:** Influenza pandemic preparedness, High pathogenic avian influenza, QIAstat-dx respiratory SARS-CoV-2 panel, Influenza a H5 detection, Influenza surveillance

## Abstract

**Introduction:**

A key factor in influenza pandemic preparedness is the ability to detect zoonotic influenza virus strains as they emerge in humans through spillover events, ideally before human-to-human transmission occurs.

**Design:**

In this study, the utility of the QIAstat-Dx syndromic device for influenza surveillance was evaluated. Bioinformatic analysis was performed on all WHO-recommended influenza Candidate Vaccine Viruses (CVVs), including the common strains recommended for the 2023–2024 influenza vaccine composition in the northern hemisphere, and 16 different H5 highly pathogenic avian influenza virus (HPAIV) and two H9N2 low pathogenic avian influenza virus (LPAIV) strains. For laboratory validation, engineered gene fragments and real HPAIV and LPAIV samples were tested using the QIAstat-Dx Respiratory SARS-CoV-2 Panel.

**Results:**

During the bioinformatic screening, common influenza strains were positive including influenza A subtypes, and all H5 HPAIV and LPAIV H9N2 were detected as Influenza A positive without subtype discrimination. In all cases, laboratory validation confirmed all bioinformatic results.

**Conclusion:**

QIAstat-Dx can detect all tested potentially zoonotic influenza A virus strains, and discriminate them from human sesonal influenza A viruses, ensuring a correct diagnosis. Any tool available for surveillance and pandemic preparedness is essential for a rapid response to reduce healthcare costs and severity of future influenza pandemics.

## 1 Introduction

Influenza A viruses are capable of crossing species barriers and circulating in a variety of different host species. In the past, zoonotic influenza A viruses have caused global human pandemics, best exemplified by the 1918 Spanish flu (Reviewed in Ref. [1]). While waterfowl are assumed to constitute the natural reservoir of influenza A viruses, intermediate animal hosts are thought to be necessary for genetic adaptation of avian influenza A viruses to humans with pandemic potential [2]. Therefore, zoonotic influenza A viruses circulating in various animal hosts, including pigs or mink, are a current public health concern and surveillance of these viruses is included in the WHO Pandemic Influenza Preparedness (PIP) Framework [3].

Since 1997, occasional events of direct transmission of H5N1 highly pathogenic avian influenza viruses (HPAIV) from poultry to humans have occurred [4,5], raising concern among public health experts [6,7]. Between 2003 and 2005, HPAIV of the H5 subtype diversified and spread to several countries in Asia, Europe, and Africa [8,9]. However, the unprecedented rapid geographic spread and high mortality rates observed in birds infected with the current panzootic H5N1 clade 2.3.4.4b have reinforced concerns that these viruses may adapt to humans [10]. Importantly, however, to date all reported human infections with H5N1 HPAIV have been the result of direct exposure to poultry, and no cases of mammal-to-human or human-to-human transmission of this virus have been documented [9,11]. In addition to H5N1 HPAIVs, other avian influenza A virus subtypes, including H9N2 and H7N7 low pathogenic avian influenza viruses (LPAIVs), have caused human infections in Hong Kong, southern China, and the Netherlands [12]. These zoonotic transmission events highlight the need to closely monitor and detect avian influenza A viruses in humans and other intermediate hosts as part of the pandemic preparedness before an adaptation to humans and onward human-to-human transmission occurs.

Following the first human infection with avian influenza A (H5N1) in 1997 [5], reports of fatal human infections [13], and the risk of possible human-to-human spread, organizations such as the World Health Organization (WHO) and the Centers of Disease Control and Prevention (CDC) have intensified pandemic preparedness planning [11,14,15,16].

Current surveillance plans for H5 HPAIV include molecular diagnostics testing using Real Time PCR (RT-PCR) for rapid detection and response. Specifically, the CDC and the WHO established various RT-PCR kits for the detection of human seasonal viruses (H1, H1N1 pdm09, and H3 strains) as well as zoonotic influenza A viruses of avian origin (H5 and H7 subtypes) [11,17,18]. Laboratory influenza diagnostics typically include only the human seasonal influenza viruses. Detection of influenza A virus without a specific subtype should be reported immediately for additional testing to determine if the infection is due to a novel influenza A virus [17,18]. We have recently shown that the commercially available multiplex kit of the QIAstat-Dx Respiratory Panel can detect various influenza A viruses, including human seasonal (H1, H1N1 pdm09, and H3), swine and avian influenza A viruses [19].

The objective of this study was to use the QIAstat-Dx Respiratory SARS-CoV-2 Panel (designated as QIAstat-Dx Respiratory Panel Plus for the US version) to enlarge the number of zoonotic strains tested in the previous publication [19] and to understand molecular detection of critical influenza strains of public health concern, such as the currently circulating panzoonotic H5N1 clade 2.3.4.4b strains. This study focused on the detectability evaluation of HPAIV and Candidate Vaccine Viruses (CVV) defined by the WHO [9,20], strains selected as CVV based on their probability to eventually jump to humans (reviewed in Ref. [21]). In this sense, all strains selected for influenza vaccine composition have been included in the analysis, covering all genetic variability of HPAIV and CVVs. To this end, CVV strains proposed by WHO for 2023–2024 season vaccine composition [20] were analyzed bioinformatically to predict their detectability, and then, artificial dsDNA gene fragments (gBlocks) were tested using the QIAstat-Dx device to confirm their detection. In addition, H5 HPAIVs and H9N2 LPAIVs described by the WHO [9] for influenza pandemic preparedness and vaccine construction were included in the bioinformatic analysis. For validation of the bioinformatic prediction, HPAIV samples were tested, and their detectability was evaluated.

## 2 Materials and methods

All CVV strains corresponding to the WHO recommendation for influenza vaccine composition for the 2023–2024 influenza season in the northern hemisphere [20] were analyzed, including egg-based and cell culture- or recombinant-based vaccines. In total, after removing repeated strains, two H3N2influenza A viruses, two H1N1 descendant influenza A viruses of the 2009 swine influenza pandemic (H1N1 pdm09) and two influenza B viruses (one of the Yamagata and one of the Victoria lineage) were analyzed (Table 1). All influenza gene sequences were collected from the GISAID database (Supplemetary Table 1). In addition, the complete list of CVV for influenza pandemic preparedness corresponding to H5 (43) and H9N2 (seven) strains were collected from the WHO report [9], and influenza gene sequences were also collected from databases (NCBI and GISAID) when available. A list of accession numbers used in this study can be found in Supplementary Table 1.

**Table 1:** Summary of QIAstat-Dx Respiratory SARS-CoV-2 Panel results for all tested CVVs samples.

Strain description					QIAstat-Dx Generic Influeza A/B detection (Ct)		QIAstat-Dx influenza A subtype detection (Ct)		
Source	Subtype (Pathogenicity)	Strain	Flu HA Clade^b^	Conc. Tested (c/mL)	Infuenza A	Infuenza B	Seasonal H1	Seasonal H3	H1N1 pdm09
WHO recommendations for quadrivalent or trivalent vaccines for use in the 2023–2024 northern hemisphere influenza season (WHO, 2023b) [20]	Influenza A H1N1 pdm09	A/Victoria/4897/2022	n/a	1.00E+07	20.8	Neg	Neg	Neg	18.6
		A/Wisconsin/67/2022	n/a	1.00E+07	22.2	Neg	Neg	Neg	20.5
	Influenza A H3N2	A/Darwin/9/2021	n/a	1.00E+07	26.8	Neg	Neg	21.9	Neg
		A/Darwin/6/2021	n/a	1.00E+07	25.5	Neg	Neg	22.8	Neg
	Influenza B Victoria lineage	B/Austria/1359417/2021	n/a	1.00E+07	Neg	22.3	Neg	Neg	Neg
	Influenza B Yamagata lineage	B/Phuket/3073/2013^a^	n/a	1.00E+07	Neg	21.1	Neg	Neg	Neg
Sample set 1	Influenza A H5N1 (HPAIV)	A/herring gull/Germany-Büsum/AI1196/2022	2.3.4.4b	9.90E+08	13.0	Neg	Neg	Neg	Neg
	Influenza A H5N1 (HPAIV)	A/herring gull/Germany-Büsum/AI7088/2022	2.3.4.4b	2.40E+09	13.5	Neg	Neg	Neg	Neg
	Influenza A H5N1 (HPAIV)	A/chicken/Germany-MV/AI01026/2022	2.3.4.4b	2.00E+08	15.6	Neg	Neg	Neg	Neg
	Influenza A H5N1 (HPAIV)	A/red knot/Ger-SH/AI00616/2022	2.3.4.4b	8.28E+06	13.6	Neg	Neg	Neg	Neg
	Influenza A H5N1 (HPAIV)	A/pigeon/Germany-NW/AI00951/2022	2.3.4.4b	7.47E+07	14.6	Neg	Neg	Neg	Neg
	Influenza A H5N1 (HPAIV)	A/black-headed gull/Germany-HH/AI01073/2022	2.3.4.4b	2.40E+09	13.9	Neg	Neg	Neg	Neg
	Influenza A H5N8 (HPAIV)	A/chicken/Germany-NW/AI03705/2021	2.3.4.4b	1.40E+08	16.1	Neg	Neg	Neg	Neg
	Influenza A H9N2 (LPAIV)	A/bat/Egypt/381OP/2017	n/a	3.00E+06	31.9	Neg	Neg	Neg	Neg
	Influenza A H9N2 (LPAIV)	A/layer chicken/Bangladesh/VP02-plaque/2016	n/a	1.00E+05	19.9	Neg	Neg	Neg	Neg
	Influenza A H7N7 (HPAIV)	A/Seal/Massachusetts/1/80	n/a	2.80E+08	15.5	Neg	Neg	Neg	Neg
	Influenza A H1N1 (Control)	A/Puerto Rico/8/34	n/a	3.33E+07	15.8	Neg	13.5	Neg	Neg
Sample set 2	Influenza A H5N1 (HPAIV)	A/duck/Ger-SH/AI05232/2022	2.3.4.4b	n/a^c^	26.4	Neg	Neg	Neg	Neg
	Influenza A H5N1 (HPAIV)	A/Bangladesh/AI02463/2022	2.3.2.1c	n/a^c^	33.3	Neg	Neg	Neg	Neg
	Influenza A H5N3 (HPAIV)	A/knot/Ger-SH/2020AI03492/2020	2.3.4.4b	n/a^c^	36.6	Neg	Neg	Neg	Neg
	Influenza A H5N4 (HPAIV)	A/buzzard/Ger-SH/AI02999/2021	2.3.4.4b	n/a^c^	36.2	Neg	Neg	Neg	Neg
	Influenza A H5N4 (HPAIV)	A/gull/Germany-SH/2021AI01498/2021	2.3.4.4b	n/a^c^	31.9	Neg	Neg	Neg	Neg
	Influenza A H5N5 (HPAIV)	A/Turkey/Germany-SH/AR425/2017	2.3.4.4b	n/a^c^	37.5	Neg	Neg	Neg	Neg
	Influenza A H5N6 (HPAIV)	A/Common pochard/Germany/AR9/2018	2.3.4.4b	n/a^c^	33.4	Neg	Neg	Neg	Neg
	Influenza A H5N8 (HPAIV)	A/Turkey/Germany-MV/AR2472/2014	2.3.4.4a	n/a^c^	37.7	Neg	Neg	Neg	Neg
	Influenza A H5N8 (HPAIV)	A/Tufted duck/Germany-SH/AR8444/2016	2.3.4.4b	n/a^c^	37.3	Neg	Neg	Neg	Neg

For further details on QIAstat-Dx cartridge runs, see Supplementary Table 2. Sample set 1 corresponds to samples collected at the Friedrich Loeffler Institute (Germany) and tested at The Institute of Virology University Hospital of Freiburg (Germany) facilities. Sample set 2 corresponds to samples collected and purified at the Friedrich Loeffler Institute (Germany) and tested at QIAGEN Barcelona (Spain) facilities. HPAI= High Pathogenic Avian Influenza. LPAI = Low Pathogenic Avian Influenza. Ct = Cycle threshold. EPF = End-point-fluorescence. Neg = Negative Result. n/a = not applicable or unknown data.

^3^H1N1 A/PuertoRico/8/1934 strain was used as a positive control of the system.

a B/Phuket/3073/2013 (B/Yamagata lineage)-like virus is only included in the quadrivalent vaccine composition.

b Only H5 HPAI IAV were described in this study according to Flu A HA gene clade classification.

c See Supplementary Table 2 for further details on the methodology used for viral load adjustments.

For the initial bioinformatic evaluation, all gene sequences were aligned using the Geneious software v10.2.6 (https://www.geneious.com), with a gap open and an extent cost of 5 and 3, respectively. The output alignment was curated manually. Official RT-PCR influenza assay oligonucleotides of the QIAstat-Dx Respiratory SARS-CoV-2 Panel, included oligonucleotides for general influenza A and B virus detection together with subtype-specific oligonulceotides for the H1, H1N1 pdm09, and H3 subtypes. For positivity evaluation, the same acceptance criteria as in Refs. [19,22,23] was used. Every screened PCR primer set was considered unspecific for the analyzed sequences if: 1) three or more mismatches were found among any individual oligonucleotide sequence and the strain gene sequences or 2) variations were placed in the three last nucleotides of the 3′-end of primers or in the 5′-end of the probe affecting the PCR amplification.

As a second step, the bioinformatic evaluation was confirmed in the laboratory. In the case of the seasonal influenza A and B virus strains recommended for the 2023–2024 northern hemisphere vaccine, artificial dsDNA sequences (gBlocks) were designed from databases (Supplemetary Table 1) and acquired from a commercial supplier (Integrated DNA Technologies (Coralville, IA, USA)). Artificial gBlock sequences corresponded to the entire genomic segments of each evaluated strain listed in Supplementary Table 1. They were prepared for testing at a concentration of 1.00E+07 copies/mL to ensure specificity, including the gene sequences targeted by the QIAstat-Dx Respiratory SARS-CoV-2 Panel.

For the HPAIV strains, virus isolates from animal samples were obtained from the Friedrich Loeffler Institute (Germany), and divided into two sets (Table 1), according to sample availability and the organization of the study. The first sample set (six H5N1, one H5N8, two H9N2, and one H7N7 virus strain) was directly tested in the Institute of Virology, University Medical Center Freiburg (Germany) under BSL3 conditions. Corresponding H1N1 control was also included in the analysis. The second set of samples (nine different H5 HPAIV strains, covering the H5N1, H5N3, H5N4, H5N5, H5N6, and H5N8 subtypes) included genomic material extracted in Friedrich Loeffler Institute using the Qiagen Viral RNA kit (Qiagen, Hilden, Germany) or NucleoMag VET kit (Macherey-Nagel GmbH & Co. KG, Düren, Germany) according to manufacturer's instructions. Extracted viral RNA was sent directly to QIAGEN facilities in Barcelona (Spain) for testing. All isolates were obtained from clinical material (swabs or tissue lysates) by inoculation via the amnio-allantoic route into embryonated chicken eggs, with a maximum of two passages in embryonated chicken eggs. Further details of the methodology used for virus isolation, culture and extraction of genomic material are provided in Supplementary Table 2.

All samples were run in a single replicate using QIAstat-Dx Respiratory SARS-CoV-2 Panel cartridges on the QIAstat-Dx Analyzer according to the manufacturer's instructions. The QIAstat-Dx Respiratory cartridge provides results for the detection of any of the 22 respiratory pathogens included in the panel in approxemately 1 h [24].

Results are shown in Supplementary Table 3 (including CT values and endpoint fluorescence) and summarized in Table 1. HPAIV and LPAIV clinical samples were run without prior dilution, yelding a positive result at the sensitivity level of the QIAstat-Dx Respiratory SARS-CoV-2 panel described in the “Instructions For Use” section [24]. Additional information on panel performance such as the use of the internal control and interpretation of results is also described.

## 3 Results

To determine the *in silico* specificity of the QIAstat-Dx Respiratory SARS-CoV-2 Panel for pandemic influenza preparedness, we retrieved the genomic sequences for common influenza strains included in the CVV report [20], and H5 HPAIV and H9N2 LPAIV strains used for vaccine development and pandemic preparedness [9] from the GISAID database (Supplementary Table 1). Because no sequences were available for the A/duck/Bangladesh/19097/2013 (H5N1, clade 2.3.2.1a) and A/Bangladesh/994/2011 (H9N2, clade G1) strains, we excluded them from further analysis. For A/bar-headed goose/Qinghai/1A/2005 (H5N1, clade 2.2), A/Egypt/2321-NAMRU3/2007 (H5N1, clade 2.2.1), A/chicken/Guiyang/1153/2016 (H5N1, clade 2.3.2.1d), and A/chicken/Bangladesh/11rs1984-30/2011 (H5N1, clade 2.3.4.2), sequences were only available for some genome segments. Importantly, the most recent zoonotic H5 HPAIV strains 2.3.4.4b and 2.3.2.1c were covered in the HPAIV CVV strain list.

After sequence alignment, oligonucleotides corresponding to all influenza A virus RT-PCR assays included in the QIAstat-Dx Respiratory SARS-CoV-2 Panel were mapped to each alignment to evaluate the potential detection of each strain analyzed. Bioinformatic evaluation of the seasonally circulating human influenza virus strains, including seasonal H1N1 and H3N2 as well as influenza B viruses of the Victoria and Yamagatha lineages, resulted in positive *in silico* detection with influenza A subtype discrimination. Specifically, all four influenza A virus strains were detected by the general influenza A RT-PCR assay, whereas the two H3N2 (A/Darwin/9/2021 and A/Darwin/6/2021) and the two H1N1 pdm09 strains (A/Victoria/4897/2022 and A/Wisconsin/67/2022) were positive by the corresponding subtype RT-PCR assay.

All human seasonal influenza A virus strains (H3N2 and H1N1 pdm09) resulted in complete coverage of the oligonucleotides of the RT-PCR assays, with a maximum of two mismatches that were never placed at the 3′ or 5’ end of the primers or the labeled probes, respectively. In addition, influenza B viruses of the Victoria and Yamagatha lineages were found to be positive for the Flu B assay with a single non-critical mismatch in the probe sequence binding region. Consistent with the *in silico* predicted high specificity of the designed oligonucleotides, laboratory testing demonstrated that all seasonal influenza A and B viruses were reliably detected by the QIAstat-Dx device (Table 1).

Next, we determined the *in silico* specificity of the oligonucleotides used for all H5 HPAIV and H9N2 LPAIV AIV CVV strains and found that the analyses were positive for the general influenza A assay, with a maximum of a single mismatch in a non-critical position. Only influenza A/chicken/Vietnam/RAHO4-CD-20-421/2020 (H5N6, clade 2.3.4.4g) strain had two non-critical mismatches in the reverse primer binding region. Importantly, the H1, H1 pdm09, and H3 subtype-specific oligonucleotides failed to map to the H5 HPAIV and H9N2 LPAIV genomes *in silico*, indicating the assay's specificity. Laboratory testing with the QIAstat-Dx device demonstrated that all HPAIV or LPAIV samples were positive with the QIAstat-Dx generic Influeza A detection and no unspecific detection with the remaining RT-PCR assays included in the testing panel was observed (Table 1 and Supplementary Table 3).

In summary, the QIAstat-Dx Respiratory SARS-CoV-2 Panel is able to reliably detect seasonal influenza A and B viruses, H9N2 LPAIVs and all tested H5 HPAIVs, including the current panzootic H5 HAPIVs of the 2.3.4.4b clade.

## 4 Discussion

The results of this study demonstrate that the QIAstat-Dx Respiratory SARS-CoV-2 Panel is capable of detecting seasonally circulating influenza A virus strains as well as all tested H9N2 LPAIV and H5 HPAIV strains of the 2.3.4.4b clade that are used for vaccine development and for pandemic influenza preparedness.

The ability to generate surveillance data during clinical diagnostic routines by detecting non-human Influenza strains and discriminating the current human subtypes is one of the key elements of syndromic testing. This added value comes at no additional cost and strengthens the current surveillance networks of reference laboratories.

A limitation of this study might be misdiagnosis due to false negative result from the influenza subtype assay included in the QIAstat-Dx Panel because of a low viral load. However, previous results [19] have shown that the QIAstat-Dx assay provides a good correlation of Ct values between RT-PCR assays (Flu A and subtypes), suggesting that human seasonal influenza A virus subtypes (H1, H1N1 pdm09 or H3) should have similar CT values in both Influenza A virus and corresponding subtype PCR assays. Therefore, a specimen that tests positive for influenza A virus but not for a specific subtype, most likely represents a zoonotic influenza A virus strain.According to official recommendations from the CDC [17] and WHO [18], such nonspecific subtype isolates of suspected zoonotic origin should be reported immediately to official agencies for further testing to confirm the presence of a novel influenza A virus.

Furthermore, because the QIAstat-Dx Respiratory SARS-CoV-2 Panel only differentiates among human H1 (seasonal and pandemic separately) subtypes at high level, after detecting the presence of any new avian influenza virus in the tested sample, further molecular analyses (whole-genome sequencing or strain-specific RT-PCRs) would be needed to correctly classify it into correct HA gene clade. This information is essential for further epidemiologic studies and phylogenetic analyses and to monitor influenza pandemic preparedness.

As a future influenza pandemic is a matter of "when" rather than "if", continuous surveillance of emerging zoonotic viruses is critical to respond as early as possible [16]. Therefore, a creation of a woldwide network of improved and rapid molecular testing systems, with cloud-connectivity for real-time data availability, are essential for the early detection and identification of zoonotic viruses. These viruses include subtypes H2, H5, H6, H7, and H9 [25], all of which can be specifically and rapidly detected by the QIAstat-Dx instrument. This makes the QIAstat-Dx instrument particularly useful for influenza surveillance and pandemic preparedness.

## CRediT authorship contribution statement

**Luis Peñarrubia:** Writing – review & editing, Writing – original draft, Supervision, Methodology, Conceptualization. **Sven Reister:** Writing – review & editing, Writing – original draft, Supervision, Methodology, Conceptualization. **Sara Jiménez-Guzmán:** Writing – review & editing, Investigation, Formal analysis, Data curation. **Roberto Porco:** Writing – review & editing, Investigation, Formal analysis, Data curation. **Clàudia Congost-Teixidor:** Writing – review & editing, Investigation, Formal analysis, Data curation. **Gemma Pueyo:** Writing – review & editing, Supervision, Conceptualization. **Carla Camprubí-Font:** Writing – review & editing, Supervision, Conceptualization. **Katariina Vara:** Writing – review & editing, Formal analysis, Conceptualization. **Maria de la Cruz Cardenosa:** Writing – review & editing, Formal analysis, Conceptualization. **Maria Contreras:** Writing – review & editing, Formal analysis, Conceptualization. **Aida Mayorgas:** Writing – review & editing, Formal analysis, Conceptualization. **Frederick van Deursen:** Writing – review & editing, Writing – original draft, Resources, Project administration, Funding acquisition, Conceptualization. **Dietrich Lueerssen:** Writing – review & editing, Resources, Project administration, Funding acquisition. **Marti Juanola-Falgarona:** Writing – review & editing, Supervision, Funding acquisition, Conceptualization. **Martin Schwemmle:** Writing – review & editing, Investigation, Formal analysis, Data curation. **Kevin Ciminski:** Writing – review & editing, Project administration, Methodology, Investigation, Formal analysis, Data curation. **Davide Manissero:** Writing – review & editing, Resources, Funding acquisition, Conceptualization.

## Ethical approval statement

No ethical approval was required to conduct this work.

## Data availability

Data associated with this study has not been deposited into a publicly available repository. All data included in this manuscript is described in the supplementary material.

## Funding Source

LP, SR, SJG, RP, CC, GP, CC, KV, MCC, MC, AM, FvD, DL, MJ and DM are QIAGEN workers. The work described in this paper was funder by the employer of the authors (QIAGEN).

## Declaration of competing interest

The authors declare the following financial interests/personal relationships which may be considered as potential competing interests:LP, SR, SJG, RP, CC, GP, CC, KV, MCC, MC, AM, FvD, DL, MJ and DM are QIAGEN workers. The work described in this paper was funded by the employer of the authors (QIAGEN).
