# Supplementary material for: Molecular diagnostics using the QIAstat-Dx syndromic device for covering avian influenza pandemic preparedness
Source: Heliyon. 2024 Nov 26;10(23):e40645. doi: 10.1016/j.heliyon.2024.e40645 (PMC11647833; doi:10.1016/j.heliyon.2024.e40645)
Supplement: Multimedia component 1 [file mmc1.docx]

WHO Candidate Virus Vaccines (Usual infecting humans Influenza strains):

| Source | Description | Strains | Sequences available | S1 (PB2) | S2 (PB1) | S3 (PA) | HA gene | S5 (NP) | NA gene | M gene | S8 (NS) |
| --- | --- | --- | --- | --- | --- | --- | --- | --- | --- | --- | --- |
| WHO CVV report^1^ | Recommendations announced for influenza vaccine composition for the 2023-2024 northern hemisphere influenza season | Egg-based vaccines | | | | | | | | | |
|  |  | - A/Victoria/4897/2022 (H1N1)pdm09-like virus | NCBI  GISAID (EPI_ISL_17102775) | n/a  EPI2447513 | n/a  EPI2447514 | n/a  EPI2447512 | OQ718989  EPI2447516 | n/a  EPI2447509 | OQ718988  EPI2447515 | n/a  EPI2447511 | n/a  EPI2447510 |
|  |  | - A/Darwin/9/2021 (H3N2)-like virus | NCBI  GISAID (EPI_ISL_16998754) | OQ719012  EPI2415906 | OQ719013  EPI2415904 | OQ719011  EPI2415901 | OQ719007  EPI2415902 | OQ719008  EPI2415903 | OQ719006  EPI2415900 | OQ719010  EPI2415907 | OQ719009  EPI2415905 |
|  |  | - B/Austria/1359417/2021 (B/Victoria lineage)-like virus | GISAID (EPI_ISL_2378894) | EPI1884968 | EPI1884969 | EPI1884967 | EPI1868375 | EPI1884964 | EPI1868374 | EPI1884966 | EPI1884965 |
|  |  | - B/Phuket/3073/2013 (B/Yamagata lineage)-like virus^2^ | GISAID (EPI_ISL_166958) | EPI1269607 | EPI552546 | EPI544265 | EPI544267 | EPI626300 | EPI544266 | EPI552545 | EPI547695 |
|  |  | Cell culture- or recombinant-based vaccines | | | | | | | | | |
|  |  | - A/Wisconsin/67/2022 (H1N1)pdm09-like virus | NCBI  GISAID (EPI_ISL_15928538) | OQ203971  EPI2224785 | OQ203972  EPI2224786 | OQ203973  EPI2224784 | OQ203974  EPI2224788 | OQ203975  EPI2224781 | OQ203976  EPI2224787 | OQ203977  EPI2224783 | OQ203978  EPI2224782 |
|  |  | - A/Darwin/6/2021 (H3N2)-like virus | NCBI  GISAID (EPI_ISL_17832065) | OQ719004  EPI2604123 | OQ719005  EPI2604121 | OQ719003  EPI2604118 | OQ718999  EPI2604119 | OQ719000  EPI2604120 | OQ718998  EPI2604117 | OQ719002  EPI2604124 | OQ719001  EPI2604122 |
|  |  | - B/Austria/1359417/2021 (B/Victoria lineage)-like virus | GISAID (EPI_ISL_2378894) | EPI1884968 | EPI1884969 | EPI1884967 | EPI1868375 | EPI1884964 | EPI1868374 | EPI1884966 | EPI1884965 |
|  |  | - B/Phuket/3073/2013 (B/Yamagata lineage)-like virus | GISAID (EPI_ISL_166958) | EPI1269607 | EPI552546 | EPI544265 | EPI544267 | EPI626300 | EPI544266 | EPI552545 | EPI547695 |

Supplementary Table 1A. WHO CVVs evaluated in this study corresponding to usual infecting humans Influenza strains. Grey strains correspond to repeated strains.

1.- WHO recommendations for quadrivalent or trivalent vaccines for use in the 2023-2024 northern hemisphere influenza season. <https://www.who.int/news/item/24-02-2023-recommendations-announced-for-influenza-vaccine-composition-for-the-2023-2024-northern-hemisphere-influenza-season>

2.- B/Phuket/3073/2013 (B/Yamagata lineage)-like virus is only included in the quadrivalent vaccine composition, based on the observed low frequency of this lineage in the field.

WHO Candidate Virus Vaccines (H5 HPAI and H9N2 LPAI strains):

| Clade | Serotype | Strain^1^ | Database | S1 (PB2) | S2 (PB1) | S3 (PA) | S4 (HA) | S5 (NP) | S6 (NA) | S7 (M) | S8 (NS) |
| --- | --- | --- | --- | --- | --- | --- | --- | --- | --- | --- | --- |
| 1 | H5N1 | A/Viet Nam/1203/2004 | NCBI | HM006756 | HM006757 | HM006758 | HM006759 | HM006760 | HM006761 | HM006762 | HM006763 |
| 1 | H5N1 | A/Viet Nam/1194/2004 | NCBI | AY651718 | AY651664 | AY651610 | AY651333 | AY651498 | AY651445 | AY651387 | AY651552 |
| 1.1 | H5N1 | A/Cambodia/R0405050/2007 | NCBI | HQ200569 | HQ200570 | HQ200571 | FJ225472 | HQ200568 | FJ225473 | HQ200572 | HQ200573 |
| 1.1.2 | H5N1 | A/Cambodia/X0810301/2013 | NCBI  GISAID (EPI_ISL_150015) | EPI537652 | EPI537653 | KF918469 | KF918470 | KF918471 | KF918472 | KF918473 | KF918474 |
| 2.1.1 | H5N1 | A/duck/Hunan/795/2002 | NCBI | CY029270 | CY029271 | CY029272 | CY028963 | CY029273 | CY029274 | CY029275 | CY029276 |
| 2.1.3.2 | H5N1 | A/Indonesia/5/2005 | NCBI | CY116643 | CY116644 | CY116645 | CY116646 | CY116647 | CY116648 | CY116649 | CY116650 |
| 2.1.3.2a | H5N1 | A/Indonesia/NIHRD11771/2011 | GISAID (EPI_ISL_98855) | EPI377957 | EPI377958 | EPI377959 | EPI341633 | EPI377960 | EPI342499 | EPI377961 | EPI377962 |
| 2.2 | H5N1 | A/bar-headed goose/Qinghai/1A/2005 | NCBI | n/a | n/a | n/a | DQ659327 | n/a | DQ659325 | n/a | n/a |
| 2.2 | H5N1 | A/chicken/India/NIV33487/2006 | NCBI | EF362425 | EF362424 | EF362423 | EF362418 | EF362421 | EF362420 | EF362419 | EF362422 |
| 2.2 | H5N1 | A/whooper swan/Mongolia/244/2005 | NCBI | GU186707 | GU186706 | GU186705 | GU186700 | GU186703 | GU186702 | GU186701 | GU186704 |
| 2.2.1 | H5N1 | A/Egypt/2321-NAMRU3/2007 | NCBI | n/a | n/a | n/a | EF535822 | n/a | FJ461635 | n/a | n/a |
| 2.2.1 | H5N1 | A/turkey/Turkey/1/2005 | NCBI | EF619975 | EF619976 | EF619979 | EF619980 | EF619977 | EF619973 | EF619978 | EF619974 |
| 2.2.1 | H5N1 | A/Egypt/N03072/2010 | NCBI  GISAID (EPI_ISL_120274) | EPI372842 | EPI372843 | EPI372841 | CY062484 | EPI372838 | CY062485 | EPI372840 | EPI372839 |
| 2.2.1.1 | H5N1 | A/Egypt/3300-NAMRU3/2008 | NCBI  GISAID (EPI_ISL_120245) | EPI372664 | EPI372665 | EPI372663 | FJ226061 | EPI372660 | FJ461658 | EPI372662 | EPI372661 |
| 2.2.1.2 | H5N1 | A/Egypt/N04915/2014 | GISAID (EPI_ISL_262572) | EPI993790 | EPI993791 | EPI993789 | EPI993793 | EPI993786 | EPI993792 | EPI993788 | EPI993787 |
| 2.3.2.1 | H5N1 | A/common magpie/Hong Kong/5052/2007 | NCBI | CY036170 | CY036171 | CY036172 | CY036173 | CY036174 | CY036175 | CY036176 | CY036177 |
| 2.3.2.1a | H5N1 | A/Hubei/1/2010 | NCBI | CY098755 | CY098756 | CY098757 | CY098758 | CY098759 | CY098760 | CY098761 | CY098762 |
| 2.3.2.1a | H5N1 | A/duck/Bangladesh/19097/2013 | n/a |  |  |  |  |  |  |  |  |
| 2.3.2.1b | H5N1 | A/barn swallow/Hong Kong/D10-1161/2010 | NCBI | KF735641 | KF735642 | KF735643 | KC357320 | KF735644 | KF735645 | KC436130 | KF735646 |
| 2.3.2.1c | H5N1 | A/duck/Viet Nam/NCVD-1584/2012 | NCBI | KF874284 | KF874285 | KF874286 | KF715205 | KF874287 | KF715206 | KF874288 | KF874289 |
| 2.3.2.1d | H5N1 | A/chicken/Guiyang/1153/2016 | NCBI |  |  |  | MT126478 |  |  |  |  |
| 2.3.4 | H5N1 | A/chicken/Hong Kong/AP156/2008 | NCBI | KF735629 | KF735630 | KF735631 | KC590650 | KF735632 | KC590649 | KF735633 | KF735634 |
| 2.3.4 | H5N1 | A/Anhui/1/2005 | NCBI | HM172438 | HM172394 | HM172342 | HM172104 | HM172254 | HM172189 | HM172159 | HM172266 |
| 2.3.4 | H5N1 | A/duck/Laos/3295/2006 | NCBI | KJ028471 | KJ028472 | KJ028473 | DQ845348 | KJ028474 | FJ169865 | KJ028475 | KJ028476 |
| 2.3.4 | H5N1 | A/Japanese white eye/Hong Kong/1038/2006 | NCBI | DQ992562 | EF123888 | EF124643 | DQ992842 | EF124341 | EF124190 | EF124039 | EF124492 |
| 2.3.4.2 | H5N1 | A/chicken/Bangladesh/11rs1984-30/2011 | NCBI | n/a | n/a | n/a | JN795924 | n/a | n/a | n/a | n/a |
| 2.3.4.2 | H5N1 | A/Guizhou/1/2013 | GISAID (EPI_ISL_135216) | EPI423876 | EPI420390 | EPI420768 | EPI420386 | EPI423875 | EPI420387 | EPI420389 | EPI420388 |
| 2.3.4.4a | H5N6 | A/Sichuan/26221/2014 | GISAID (EPI_ISL_163493) | EPI533585 | EPI533586 | EPI533587 | EPI533583 | EPI533588 | EPI533584 | EPI533589 | EPI533590 |
| 2.3.4.4b | H5N8 | A/Astrakhan/3212/2020 | GISAID (EPI_ISL_1038924) | EPI1846958 | EPI1846959 | EPI1846960 | EPI1846961 | EPI1846962 | EPI1846963 | EPI1846964 | EPI1846965 |
| 2.3.4.4c | H5N8 | A/gyrfalcon/Washington/41088-6/2014 | NCBI | KP307981 | KP307982 | KP307983 | KP307984 | KP307985 | KP307986 | KP307987 | KP307988 |
| 2.3.4.4e | H5N6 | A/duck/Hyogo/1/2016 | GISAID (EPI_ISL_239351) | EPI866704 | EPI866706 | EPI866707 | EPI866708 | EPI866709 | EPI866710 | EPI866711 | EPI866712 |
| 4 | H5N1 | A/goose/Guiyang/337/2006 | NCBI | DQ992579 | EF123905 | EF124660 | DQ992765 | EF124358 | EF124207 | EF124056 | EF124509 |
| 7.1 | H5N1 | A/chicken/Vietnam/NCVD-016/2008 | GISAID (EPI_ISL_28830) | EPI351595 | EPI351596 | EPI351594 | EPI173704 | EPI351591 | EPI173705 | EPI351593 | EPI351592 |
| 7.1 | H5N1 | A/chicken/Vietnam/NCVD-03/2008 | GISAID (EPI_ISL_80638) | EPI349845 | EPI349846 | EPI349844 | EPI284473 | EPI349841 | EPI349847 | EPI349843 | EPI349842 |
| 2.3.4.4h | H5N6 | A/Guangdong/18SF020/2018 | GISAID (EPI_ISL_337274) | EPI1352810 | EPI1352811 | EPI1352809 | EPI1352813 | EPI1352806 | EPI1352812 | EPI1352808 | EPI1352807 |
| 2.3.2.1a | H5N1 | A/duck/Bangladesh/17D1012/2018-like* | GISAID (EPI_ISL_331119) | EPI1318143 | EPI1318144 | EPI1318142 | EPI1318146 | EPI1318139 | EPI1318145 | EPI1318141 | EPI1318140 |
| 2.3.2.1f | H5N1 | A/chicken/Ghana/20/2015-like* | GISAID (EPI_ISL_223929) | EPI770638 | EPI770639 | EPI770637 | EPI770632 | EPI770634 | EPI770633 | EPI770636 | EPI770635 |
| 2.3.4.4d | H5N6 | A/Hubei/29578/2016-like* | GISAID (EPI_ISL_256213) | EPI961176 | EPI961177 | EPI961178 | EPI961183 | EPI961180 | EPI961181 | EPI961182 | EPI961179 |
| 2.3.4.4b | H5N6 | A/Fujian-Sanyuan/21099/2017-like* | GISAID (EPI_ISL_304404) | EPI1202731 | EPI1202730 | EPI1202724 | EPI1202729 | EPI1202726 | EPI1202727 | EPI1202728 | EPI1202725 |
| 2.3.4.4f | H5N6 | A/chicken/Vietnam/NCVD-15A59/2015* | NCBI | KY171722 | KY171723 | KY171724 | KY171725 | KY171726 | KY171727 | KY171728 | KY171729 |
| 2.3.4.4g | H5N6 | A/chicken/Vietnam/RAHO4-CD-20-421/2020-like* | GISAID (EPI_ISL_1379443) | EPI1853935 | EPI1853936 | n/a | EPI1853938 | EPI1853932 | EPI1853937 | EPI1853934 | EPI1853933 |
| 2.3.4.4b | H5N1 | A/chicken/Ghana/AVL-763_21VIR7050-39/2021-like* | GISAID (EPI_ISL_16997921) | EPI2415852 | EPI2415853 | EPI2415851 | EPI2415855 | EPI2415848 | EPI2415854 | EPI2415850 | EPI2415849 |
| 2.3.4.4b | H5N1 | A/American Wigeon/South Carolina/22-000345-001/2021* | GISAID (EPI_ISL_17008863) | EPI2416934 | EPI2416935 | EPI2416933 | EPI2416937 | EPI2416930 | EPI2416936 | EPI2416932 | EPI2416931 |
| G1 | H9N2 | A/Hong Kong/1073/99 | NCBI | NC_004910 | NC_004911 | NC_004912 | NC_004908 | NC_004905 | NC_004909 | NC_004907 | NC_004906 |
| Y280/G9 | H9N2 | A/chicken/Hong Kong/G9/97 | NCBI | AF156430 | AF156416 | AF156444 | AF156373 | AF156402 | AF156391 | AF156458 | AF156472 |
| G1 | H9N2 | A/Hong Kong/33982/2009 | NCBI | KF188313 | KF188314 | KF188315 | KF188316 | KF188317 | KF188318 | KF188319 | KF188320 |
| G1 | H9N2 | A/Bangladesh/994/2011 | n/a | n/a | n/a | n/a | n/a | n/a | n/a | n/a | n/a |
| Y280/G9 | H9N2 | A/Hong Kong/308/2014 | GISAID (EPI_ISL_153064) | EPI498034 | EPI498035 | EPI498033 | EPI498037 | EPI498030 | EPI498036 | EPI498032 | EPI498031 |
| Y280/G9 | H9N2 | A/Anhui-Lujiang/39/2018 | GISAID (EPI_ISL_330737) | EPI1315823 | EPI1315824 | EPI1315825 | EPI1315830 | EPI1315827 | EPI1315828 | EPI1315829 | EPI1315826 |
| G1 | H9N2 | A/Oman/2747/2019-like* | GISAID (EPI_ISL_353983) | EPI1431477 | EPI1431478 | EPI1431476 | EPI1431480 | EPI1431473 | EPI1431479 | EPI1431475 | EPI1431474 |

Supplementary Table 1B. WHO CVVs evaluated in this study corresponding to H5HPAI and H9N2 LPAI.

*Strains corresponding to vaccines under construction.

1.- WHO recommendations for vaccines for pandemic preparedness: <https://cdn.who.int/media/docs/default-source/influenza/who-influenza-recommendations/vcm-northern-hemisphere-recommendation-2023-2024/20230224_zoonotic_recommendations.pdf?sfvrsn=38c739fa_4>
