# Supplementary material for: Molecular diagnostics using the QIAstat-Dx syndromic device for covering avian influenza pandemic preparedness
Source: Heliyon. 2024 Nov 26;10(23):e40645. doi: 10.1016/j.heliyon.2024.e40645 (PMC11647833; doi:10.1016/j.heliyon.2024.e40645)
Supplement: Multimedia component 2 [file mmc2.docx]

Supplementary Table 2. methodology for virus isolation, culture, genomic material extraction.

| # Sample set | Virus isolation and culture | Genomic material extraction |
| --- | --- | --- |
| Sample set 1 | The HPAIVs A/herring gull/Germany-Büsum/AI1196/2022, A/herring gull/Germany-Büsum/AI7088/2022, A/chicken/Germany-MV/AI01026/2022, A/red knot/Ger-SH/AI00616/2022, A/pigeon/Germany-NW/AI00951/2022, A/black-headed gull/Germany-HH/AI01073/2022, A/chicken/Germany-NW/AI03705/2021, and bat-derived H9N2 A/bat/Egypt/381OP/2017 isolate were propagated in embryonated SPF-chicken eggs at 37°C. At 5 days post infection the allantoic fluid was harvested and used as virus stock.  The A/layer chicken/Bangladesh/VP02-plaque/2016 isolate was propagated on LMH cells in infection medium (Dulbecco’s modified Eagle’s medium, Gibco, Thermo Fisher Scientific) containing 0.2% BSA and 100 U penicillin and 100 µg streptomycin µL^-1^ for 2 days at 37°C and 5% CO_2_. Infectious cell supernatant was used as a virus stock.  The A/Seal/Massachusetts/1/80 and A/Puerto Rico/8/34 viruses were propagated on MDCK II cells in infection medium for 2 days at 37°C and 5% CO_2_. Infectious cell supernatant was used as a virus stock. | No genomic material extraction is needed prior QIAstat-Dx usage.  To evaluate the specificity of the QIAstat-Dx Respiratory SARS-CoV-2 Panel cartridges for detection of HPAIV and LPAIV strains, 300 µL of undiluted infectious virus stock were used for analysis. |
| Sample set 2 | All isolates have been obtained from clinical material (swabs or tissue lysates) by inoculation via the amnio-allantoic route into embryonated chicken eggs and propagation at 37°C for up to 5 days. The isolates have received a maximum of two passages in the allantoic cavity of embryonated chicken eggs at 37°C until embryonic death became evident (daily candling of eggs), usually between days 2-4 after inoculation. | Undiluted amnio-allantoic fluid (AAF) was used for extraction; infectivity titres typically exceeded 1E+08 TCID_50_ per mL of AAF. The Qiagen Viral RNA kit or Macherey & Nagel NucleoMag kits have been used for manual extraction following the recommendations of the manfacturers. No DNAse step had been applied. Extracted RNA was further diluted at a ratio of 1:200 in RNA Safe buffer (50 ng/µL carrier polyA-RNA (RNA-homopolymer, #10108626001; Roche Diagnostic), 0.05% Tween 20, 0.05% Sodium azide) to finally yield CT values of 25-30 per 5 µL in a generic influenza A virus real time RT PCR targeting the M gene segment (Hassan et al., 2022; PMID 35216008). |

References: Hassan KE, Ahrens AK, Ali A, El-Kady M, Hafez HM, Mettenleiter TC, Beer M, Harder T. 2022. Improved Subtyping of Avian Influenza Viruses Using an RT-qPCR-Based Low Density Array: ‘Riems Influenza a Typing Array’, Version 2 (RITA-2). Viruses 2022, 14, 415. <https://doi.org/10.3390/v14020415>.
